# Ser-653-Asn substitution in the acetohydroxyacid synthase gene confers resistance in weedy rice to imidazolinone herbicides in Malaysia

**DOI:** 10.1371/journal.pone.0227397

**Published:** 2020-09-14

**Authors:** Rabiatuladawiyah Ruzmi, Muhammad Saiful Ahmad-Hamdani, Norida Mazlan

**Affiliations:** 1 Department of Crop Science, Faculty of Agriculture, Universiti Putra Malaysia, Selangor, Malaysia; 2 Laboratory of Climate-Smart Food Crop Production, Institute of Tropical Agriculture and Food Security, Universiti Putra Malaysia, Selangor, Malaysia; 3 Department of Agriculture Technology, Faculty of Agriculture, Universiti Putra Malaysia, Selangor, Malaysia; Louisiana State University College of Agriculture, UNITED STATES

## Abstract

The continuous and sole dependence on imidazolinone (IMI) herbicides for weedy rice control has led to the evolution of herbicide resistance in weedy rice populations across various countries growing IMI herbicide-resistant rice (IMI-rice), including Malaysia. A comprehensive study was conducted to elucidate occurrence, level, and mechanisms endowing resistance to IMI herbicides in putative resistant (R) weedy rice populations collected from three local Malaysian IMI-rice fields. Seed bioassay and whole-plant dose-response experiments were conducted using commercial IMI herbicides. Based on the resistance index (RI) quantification in both experiments, the cross-resistance pattern of R and susceptible (S) weedy rice populations and control rice varieties (IMI-rice variety MR220CL2 and non-IMI-rice variety MR219) to imazapic and imazapyr was determined. A molecular investigation was carried out by comparing the acetohydroxyacid synthase (AHAS) gene sequences of the R and S populations and the MR220CL2 and MR219 varieties. The AHAS gene sequences of R weedy rice were identical to those of MR220CL2, exhibiting a Ser-653-Asn substitution, which was absent in MR219 and S plants. *In vitro* assays were conducted using analytical grade IMI herbicides of imazapic (99.3%) and imazapyr (99.6%) at seven different concentrations. The results demonstrated that the AHAS enzyme extracted from the R populations and MR220CL2 was less sensitive to IMI herbicides than that from S and MR219, further supporting that IMI herbicide resistance was conferred by target-site mutation. In conclusion, IMI resistance in the selected populations of Malaysian weedy rice could be attributed to a Ser-653-Asn mutation that reduced the sensitivity of the target site to IMI herbicides. To our knowledge, this study is the first to show the resistance mechanism in weedy rice from Malaysian rice fields.

## Introduction

Herbicide resistance is the ability of plants to inheritably survive and reproduce following herbicide application at a rate that would normally kill the wild/susceptible type plants [1–3]. Resistance may develop naturally in plants owing to herbicide selection pressure or may be induced by genetic engineering or mutagenesis [1–3], and it is commonly associated with herbicide rates higher than the recommended dose. Notably, several plant species are naturally able to tolerate certain herbicides; however, this tolerance is limited to a normal/recommended herbicide dose [1, 2]. Herbicide resistance mechanisms have been broadly categorized into two types, namely, target site resistance (TSR) and non-target site resistance (NTSR) [4]. In TSR, the target enzymes show reduced sensitivity to the herbicide owing to mutation(s) at the site of action that prevents the herbicide from binding to the enzyme, which may lead to altered epigenetic regulation in some cases, such as overexpression of the enzyme or gene amplification [4–7]. In contrast, NTSR is associated with one or combination of mechanisms resulting in rapid herbicide detoxification, herbicide sequestration, decreased herbicide penetration rate, and reduced herbicide translocation [4, 8]. Of the 512 herbicide resistance cases reported worldwide, resistance to acetohydroxyacid synthase (AHAS) inhibitors is the most frequent (165 cases) [9], and amino acid substitutions within the AHAS gene represent the most common mechanism endowing resistance to AHAS inhibitors in weed species [10, 11].

Acetohydroxyacid synthase (AHAS, EC 2.2.1.6) is a biosynthetic enzyme that catalyzes the *de novo* step in the synthesis of branched-chain amino acids, *viz*, valine, leucine, and isoleucine [12]. These amino acids are the building blocks of proteins and are essential for the development and growth of new cells in plants. Their absence can inhibit plant root and shoot growth, ultimately leading to plant death [13]. The AHAS enzyme is the target of many commercial herbicides, including imidazolinone (IMI), with more than 50 AHAS-targeting herbicides in commercial use worldwide [14].

Herbicide-tolerant crops, particularly IMI-resistant rice varieties (e.g., Clearfield^®^ Rice Production System or CPS), are presently the preferred chemical-based method for controlling weedy rice in Malaysia. Previous chemical methods used for controlling weedy rice were not effective because of genetic similarity with the conventionally cultivated rice varieties. The IMI AHAS inhibitors imazapic and imazapyr were selected for development of the IMI herbicide-resistant rice technology to control weedy rice in Malaysia. In Malaysia, the IMI-resistant rice (hereafter IMI-rice) varieties are the only commercialized herbicide-resistant rice. The introduction of non-transgenic IMI-rice varieties in Malaysian rice fields in late 2010 received a positive response, with continued cultivation of these varieties by the country’s rice growers. However, evolution of resistance to IMI herbicides has been reported in weedy rice populations in recent years [15–17]. This phenomenon has greatly affected the staple food industry in Malaysia and has raised concerns regarding the potential evolution of herbicide resistance in these weedy populations.

Weedy rice (*Oryza sativa* L.) has been the most problematic weed in Malaysian rice fields since the introduction of the direct seeding method in the early 1980s [18]. It is closely related to cultivated rice, making it difficult to control using selective herbicides [19]. The use of herbicide-resistant rice cultivars, such as IMI-rice varieties, were advocated by researchers and government in 2010, has been a successful strategy for the selective control of weedy rice in cultivated rice fields. However, over-reliance on the same weedy rice management method by rice growers has resulted in the evolution of herbicide resistance in weedy rice [15, 16, 20]. Another concern is on the occurrence of hybridization between IMI-rice and weedy rice, and the potential crosses among the weedy rice variants [21–25], as they may affect the future of herbicide-resistant rice technology. Engku et al. [17] indicated the potential of hybridization between IMI-rice and Malaysian weedy rice through pollen mediated gene flow. However, no study has been conducted in Malaysia to establish this relationship.

Currently, weedy rice represents the eighth herbicide-resistant weed species in Malaysian rice fields [9] and has been recognized as the most problematic weed in rice growing areas of Malaysia [26]. Recently, rice growers who have been cultivating IMI-rice for more than eight planting seasons in Selangor, Perak, Pulau Pinang, Kedah, and Perlis states of Malaysia have reported reduced efficacy of IMI herbicides to control weedy rice and other weed species in their fields. More recent findings have confirmed the evolution of IMI resistance in weedy rice populations of Pendang, Kedah, with weedy rice exhibiting 67-fold greater resistance than the susceptible population [16]. Presently, there is a high risk of the evolution of IMI resistance in Malaysian weedy rice populations. Thus, the objectives of the present study were (1) to quantify the occurrence and level of resistance to AHAS-targeting IMI herbicides in putative resistant weedy rice populations, (2) to identify the AHAS gene mutations conferring TSR to AHAS-targeting IMI herbicides in the confirmed resistant weedy rice populations, and (3) to investigate the existence of possible NTSR mechanisms in the resistant weedy rice individuals by comparing the AHAS enzyme activity and its inhibition by IMI herbicides.

## Materials and methods

### Seed source

Putative resistant (R) weedy rice populations were collected from IMI-rice fields, from three townships on the border of Kedah and Perlis states of Malaysia: Kampung Simpang Sanglang, Perlis (hereafter referred to as population A); Kampung Behor Mentalon, Perlis (population B); and Kampung Sungai Kering, Kedah (population C). Seeds from the herbicide-susceptible (S) population were obtained from Kampung Tanjong, Kedah, from fields not previously exposed to IMI herbicides. The IMI-rice variety MR220CL2 and non-IMI-rice variety MR219 were used as positive controls, and their seeds were obtained from the Department of Agriculture, Malaysia. The sampling locations for putative R populations were selected based on the complaints received from IMI-rice growers regarding the ineffectiveness of IMI herbicides to control weedy rice in their rice fields. It must be noted that Kedah and Perlis were among the first states to adopt IMI-rice cultivation following its introduction in 2010. Seeds were surveyed following the zig-zag pattern during the first planting season of the year, i.e., February 2017. Seeds were collected approximately 1 week before harvest. The collected seeds were placed in a paper bag and immediately transported to the laboratory. Seeds were manually threshed, cleaned, and sorted to remove contaminants, following which they were air-dried until the moisture content was below 14%. The seeds were finally labeled, sealed in a plastic bag, and stored in a refrigerator at 4°C until further analyses.

### Seed bioassay

Seeds from R and S weedy rice populations were treated with a commercial seed growth enhancer (ZAPPA^®^ Plus, containing hydrogen peroxide, sulfuric acid, and formaldehyde, composition ratio unspecified; Diversatech (M) Sdn Bhd, Malaysia) for 24 h to induce seed germination, following which they were drained and incubated at room temperature (24–26°C) until ~1 mm of the radicle had emerged. Twenty uniformly pre-germinated seeds were placed in disposable plastic Petri dishes (9 cm diameter) containing two sheets of Whatman No. 1 filter paper and 5 mL aliquots of an aqueous solution of commercial IMI herbicides (OnDuty™ WG, BASF Malaysia Sdn. Bhd.) containing the Tenagam surfactant, at seven different concentrations: 0 (distilled water only), 0.19, 0.39, 0.77, 1.55, 3.08, and 6.16 mg L^-1^ (where 0.77 mg L^-1^ represents the recommended rate). The Petri dishes were transferred to a growth chamber and incubated at 30/20°C (day/night temperature) with a 12/12 h day/night cycle under fluorescent light (8500 lx). The relative humidity ranged between 30% and 50%. The lids of the Petri dishes were not sealed to allow gas exchange and avoid anaerobic conditions. Distilled water (5 mL) was added daily, beginning 24 h after the addition of the IMI herbicides to the Petri dishes, to maintain the moisture level. The germinated seedlings were counted 14 days after the herbicide treatment, and shoot and root lengths were measured from the point of attachment of the seed to the tip of the epicotyl and hypocotyl, respectively. The experiment was performed twice.

### Whole-plant dose-response

The whole-plant dose-response experiment was conducted in glasshouses at Farm 2, Universiti Putra Malaysia, from March to August 2016. Seeds from R and S weedy rice populations were treated with a commercial seed growth enhancer (ZAPPA^®^ Plus, Diversatech (M) Sdn Bhd) for 24 h, drained, and incubated at room temperature until germination. Ten emerged seedlings (radicle approximately 1–2 cm) were transferred to 37 cm × 30 cm × 7.5 cm plastic trays filled 2/3 with commercial paddy soil and grown in flooded condition (2–5 cm). Plants were maintained following the guidelines outlined in ‘Manual Teknologi Penanaman Padi Lestari’ (Manual for Sustainable Rice Cultivation Technology), a manual provided by the Malaysian Agricultural Research and Development Institute (MARDI) for rice cultivation and widely used by extension officers and farmers in Malaysia. At the 1–2-leaf stage, all seedlings were treated with a commercial IMI herbicide mix containing imazapic and imazapyr (OnDuty™ WG, BASF Malaysia Sdn. Bhd.) with Tenagam surfactant, at seven different rates: 0 (water only), 38.5, 77.0, 154.0, 308.0, 616.0, and 1232.0 g a.i. ha^-1^ (where 154.0 g a.i. ha^-1^ represents the recommended rate). The herbicide was applied using a compression-type sprayer with a detachable flat fan nozzle, delivering 200 L ha^-1^ at a spray pressure of 150 kPa. Average day/night temperatures of 33/24°C were maintained during the experiment, resembling the actual rice field conditions. Surviving plants were counted and visually assessed 21 days after the herbicide treatment. Surviving plants that continued to produce new shoots, or tillers, following the herbicide treatment were regarded as confirmed R plants, and those displaying severe leaf chlorosis, desiccation, retarded growth or no new active growth, and eventual plant death were considered as S plants. The positive control varieties were assessed in a similar manner. Both treated and untreated plants were cut 1 cm above the ground, dried at 65°C for 72 h, and weighed. The dry weight of all plants (dead and alive) was recorded for each population. All R weedy rice plants surviving herbicide application at and above the recommended rate, as well as the shoot materials from untreated S plants, MR220CL2, and MR219, were allowed to regrow, and the newly produced shoots and leaves were sampled and stored at -80°C for the AHAS gene mutation study. The experiment was replicated twice.

### DNA extraction and amplification of AHAS gene fragment

Post-harvest shoot materials from the R weedy rice populations (populations A, B, and C) and the untreated S weedy rice plants, MR220CL2, and MR219, were sampled for genomic DNA extraction and gene sequencing for the AHAS gene mutation study. Fifty-two leaf samples (~100 mg per plant) from R weedy rice plants and three samples each from S weedy rice, MR220CL2, and MR219 plants were extracted using a modified cetyltrimethylammonium bromide protocol [27]. The nucleotide sequence of the AHAS gene fragment, including the potential mutation sites, was amplified by polymerase chain reaction (PCR), using the forward primer 5ʹ-GTAAGAACCACCAGCGACACC-3ʹ and the reverse primer 5ʹ-GATGCATATGCCTACGGAAAAC-3ʹ [28], to study the molecular basis of IMI resistance in weedy rice populations and control rice varieties. PCR reactions were performed in a 15 μL volume, containing 7.5 μL PCR MyTaq Red Mix (Bioline, GmbH, Germany), 1.0 μL genomic DNA template (70 ng μL^-1^), 1 μL of each primer (10 mM), and 4.5 μL deionized water. PCR was performed in a heated lid PCR machine (Thermal Cycler T100, Bio-Rad, Hercules, CA, USA), with the following cycling conditions: denaturation at 95°C for 5 min, followed by 34 cycles of 30 s at 95°C, annealing at 67°C for 30 s, and elongation at 72°C for 120 s, followed by a final elongation step at 72°C for 5 min. The amplified PCR products were resolved on 1.2% agarose gels stained with FloroSafe DNA Stain (Axil Scientific Pte Ltd, Singapore), using a 1 kb DNA ladder (Fermentas, Thermo Scientific) as the reference. Unpurified PCR products were immediately sent to a commercial sequencing service (First BASE Laboratories Sdn. Bhd.) for purification, gel extraction using regular agarose, and DNA sequencing. The AHAS sequence chromatograms for all samples were visualized using DNA Baser Assembler v5.15.0. The same software was used to align and compare the obtained nucleotide sequences, which were then translated to the respective amino acid sequences using BLASTX (http://www.ncbi.nlm.nih.gov/). All amino acid sequences were standardized to the *Arabidopsis* sequence (NM114714).

### AHAS enzyme inhibition assay

All herbicide-treated parent plants from the R weedy rice populations and untreated plants from the S and control rice populations sampled for the gene mutation study were allowed to regrow for seed production. Seeds were harvested, and the progeny lines of R, S, and control rice varieties were regrown in the glasshouse until the 3–4-leaf stage. Leaf tissues from these progeny lines were used for the *in vitro* AHAS activity and inhibition assays, performed according to the method described by Yu et al. [29], with some modifications. The protein concentration of the extract was determined using a spectrophotometer following the Bradford method, and the extract was immediately used for the inhibition assay. Certified analytical standards of imazapic (99.3%) and imazapyr (99.6%) herbicides were incubated with partially purified enzyme extracts for 60 min. Then, 40 μL 6N H_2_SO_4_ was added to cease the reaction, and the mixture was incubated again at 60°C for 15 min to facilitate the conversion of acetolactate to acetoin. The acetoin, thus, formed was quantified colorimetrically at 530 nm. Enzyme assays were performed with three independent extractions, and protein concentration was measured four times for each sample.

### Statistical analyses

The seed bioassay was performed in a completely randomized design, and the whole-plant dose-response experiment was performed in a completely randomized block design, with four replications per treatment for both experiments. Seed germination rate, shoot length, root length, plant survival rate, shoot dry weight, and data from the AHAS enzyme inhibition assay were expressed as percentages of the untreated controls. All analyses were conducted using SigmaPlot Version 11.0 (Systat Software Inc., GmbH, Germany). A non-linear regression using the logistic response equation (Eq 1) proposed by Knezevic et al. [30] was used to obtain the dose-response curve, as follows:

Y=c+{d–c/1+exp[b(logx–loge)]} (1)

where c is the lower limit, d is the upper limit, b is the slope, and ED_50_ is the dose required for a 50% effect.

In the regression equation, the dose of herbicide was the independent variable (x), and the percentage of the control (plant survival) was the dependent variable (y). The fitted equations were used to calculate the amount of herbicide resulting in a 50% reduction in plant dry weight (GR_50_ value), 50% reduction in plant survival (LD_50_ value), or 50% inhibition of the AHAS activity *in vitro* (I_50_).

## Results

### Seed bioassay

**Fig 1:**
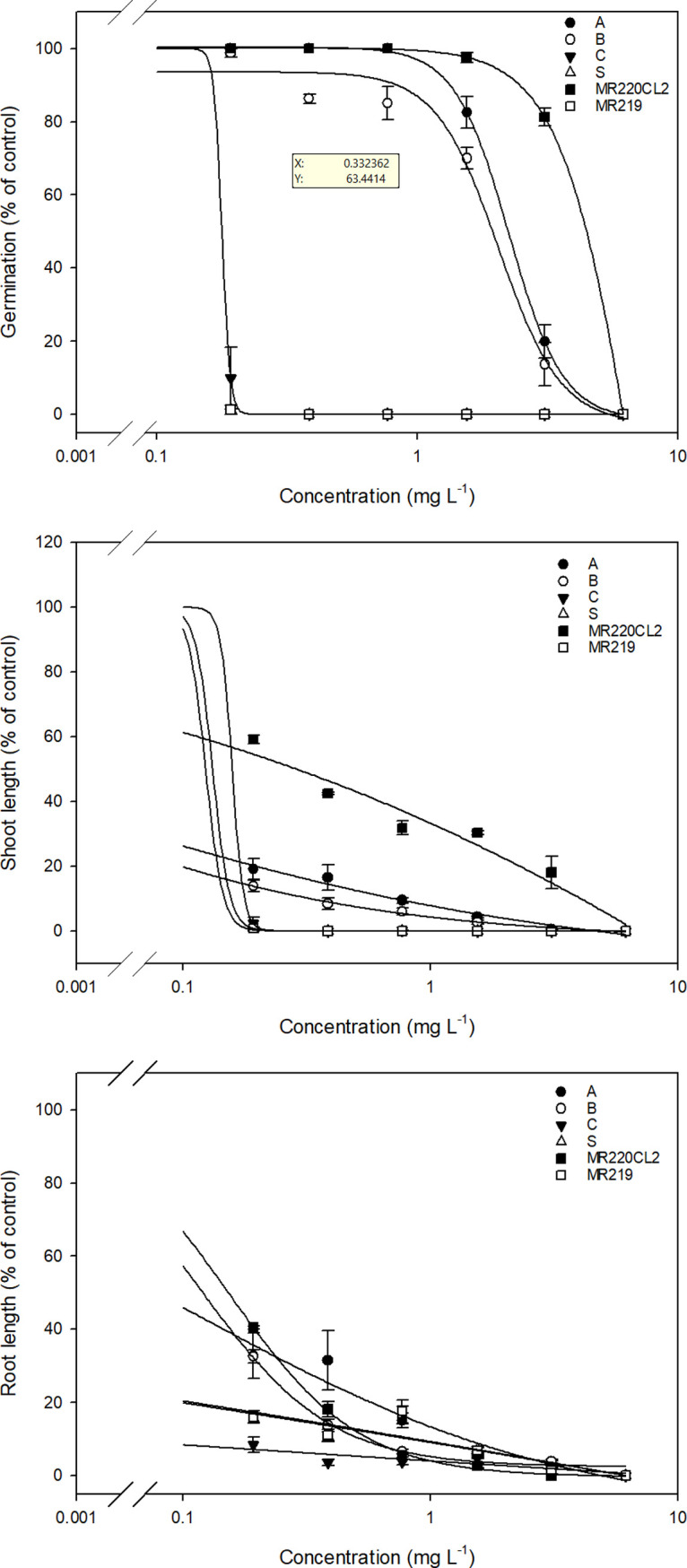
**A.** Germination rate (% of control) of weedy rice populations and control rice varieties 14 days after treatment in the seed bioassay. **B.** Shoot length (% of control) of weedy rice populations and control rice varieties 14 days after treatment in the seed bioassay. **C.** Root length (% of control) of weedy rice populations and control rice varieties 14 days after treatment in the seed bioassay.

The IMI herbicide resistance level in the weedy rice populations was quantified based on the resistance index (RI) value of the herbicide rate resulting in 50% mortality (LD_50_). The resistance level was categorized as high (>15), moderate (7–15), low (≥2 to 6), and sensitive (<2), relative to that in Iwakami et al. [31] and Merotto et al. [32]. Table 1 shows the RI values of the assessed populations of weedy rice and control rice varieties based on the seed germination percentage obtained in the seed bioassay. The MR220CL2 variety was highly resistant to pre-emergence herbicide application, with an LD_50_ RI value 50 times higher than that of the S population. Population A exhibited high herbicide resistance; however, it was considerably lower than that of MR220CL2. Population B exhibited moderate herbicide resistance, whereas populations C, S, and MR219 were equally sensitive to the applied herbicides, exhibiting no germination even at a concentration of 0.39 mg L^-1^ (half the recommended rate) (Fig 1A).

**Table 1:** Dose required to cause 50% mortality (LD_50_) and Resistance Index (RI) of weedy rice populations and control rice varieties in the seed bioassay. Standard errors are indicated in parentheses.

Population	LD_50_ (mg L^-1^)	RI (LD_50_ R/LD_50_ S)	Resistance level
A	2.25 (0.56)	16.35	High
B	2.05 (7.31)	14.92	Moderate
C	0.18 (0)	1.30	Sensitive
S	0.14 (0)	-	Sensitive
MR220CL2	7.07 (0.15)	50.50	High
MR219	0.14 (0)	1.07	Sensitive

**Fig 2:**
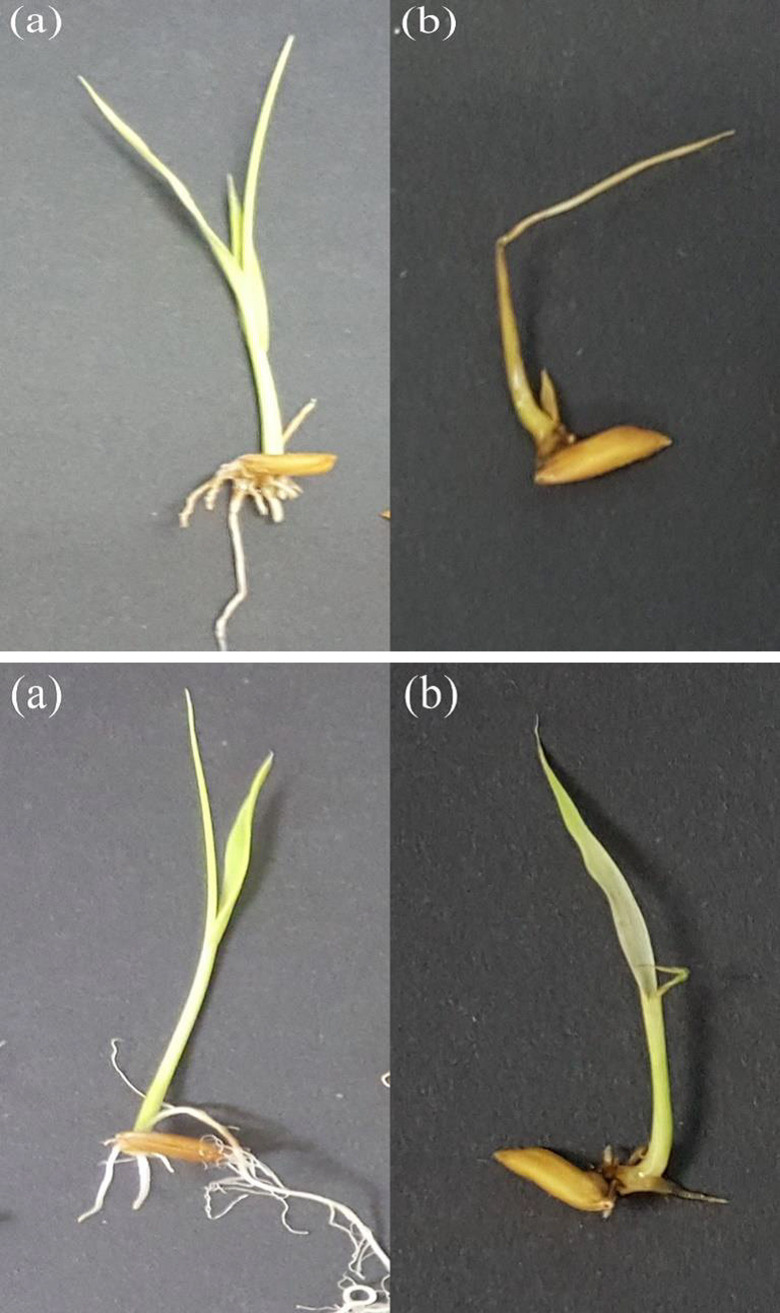
**A.** Desiccation symptoms on the shoots of weedy rice and control rice seedlings treated with imidazolinone (IMI) herbicides (b) in comparison with the untreated control (a). **B.** Blackening of roots in weedy rice and control rice seedlings treated with imidazolinone (IMI) herbicides (b) in comparison with the untreated control (a).

The effect of IMI herbicides on shoot length was considerably more severe than on the root length for all weedy rice populations and the MR219 variety (Fig 1B and 1C). Conversely, the roots of MR220CL2 were more sensitive to herbicide application than the shoots. Overall, no shoot or root growth was recorded at 6.16 mg L^-1^ herbicide concentration (eight times higher than the recommended rate) for any population. Similar to the germination rate, both shoot and root growth were inhibited in populations A and B at 6.16 mg L^-1^ herbicide concentration. However, the magnitude of growth inhibition was different for shoots and roots in the two populations, which presented 86–100% shoot inhibition compared with 68–100% root inhibition (Table 1). For population C, the shoots were completely (100%) inhibited at the recommended rate (0.77 mg L^-1^), whereas the roots elongated at all herbicide concentrations lower than four-times the recommended rate (i.e., below 3.08 mg L^-1^). A similar growth pattern was recorded for the S population and MR219 plants, where the shoots were completely inhibited at only half the recommended rate (0.39 mg L^-1^), whereas the roots grew with herbicide application four-times the recommended rate (3.08 mg L^-1^). In contrast, the shoots of MR220CL2 were still growing at the dose where the roots of the same variety were completely inhibited (3.08 mg L^-1^). The effects of IMI herbicides on shoot and root lengths of all assessed weedy rice populations and control rice varieties were visible as soon as the seeds germinated and lasted for 14 days of incubation. The treated seeds showed severe symptoms, e.g., stunted epicotyl and hypocotyl growth, which led to the inhibition of shoot and root elongation when compared with that of the untreated control seeds (Fig 2A and 2B).

### Whole-plant dose-response

**Fig 3:**
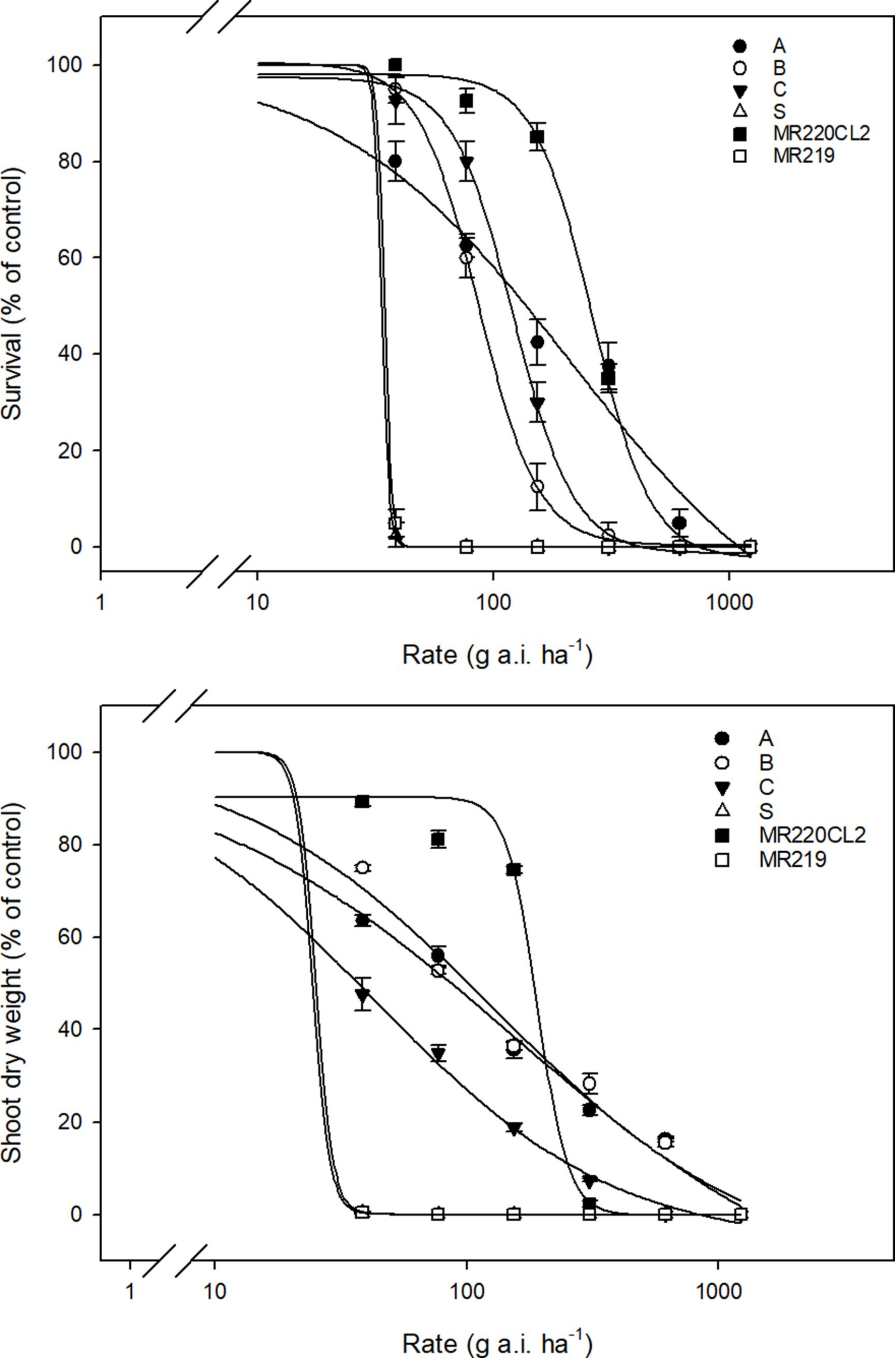
**A.** Survival (% of control) of weedy rice populations and control rice varieties 21 days after treatment in the whole-plant dose-response experiment. **B.** Shoot dry weight (% of control) of weedy rice populations and control rice varieties 21 days after treatment in the whole-plant dose-response experiment.

The weedy rice populations, and the non-IMI- and IMI-rice varieties exhibited differential sensitivity to increasing IMI herbicide rates (Fig 3A and 3B). A slightly different resistance pattern was noted compared with that in the seed bioassay, and differences between LD_50_ and GR_50_ values (Table 2) were observed at the whole-plant level. MR220CL2 was the most resistant to IMI herbicides, which was consistent with the results of the seed bioassay. It was also observed that the LD_50_ RI of all R populations, except population C, and MR220CL2 at the whole-plant level was lower than the LD_50_ RI values recorded in the seed bioassay. Based on the LD_50_ value, MR220CL2 exhibited moderate resistance, whereas populations A and B exhibited low herbicide resistance at the whole-plant level. Both MR219 and the S population were equally sensitive to the applied herbicide. However, population C exhibited low resistance, based on LD_50_, which was different from its GR_50_ and LD_50_ in the seed bioassay that corresponded to herbicide sensitivity. At the highest application rate (1232 g a.i. ha^-1^; 16 times higher than the recommended rate), no survival (100% plant shoot inhibition) was recorded in any of the assessed populations or control varieties. The non-IMI-rice variety MR219 can be considered as a susceptible positive control in this experiment, whereas MR220CL2 can be acknowledged as the resistant positive control, exhibiting the highest LD_50_ and GR_50_ values.

**Table 2:** Dose required to cause 50% mortality (LD_50_) and 50% reduction in the shoot dry weight (GR_50_), and the Resistance Index (RI) of weedy rice populations and control rice varieties in the whole-plant dose-response experiment. Standard errors are indicated in parentheses.

Population	LD_50_ (g a.i. h^-1^)	RI (LD_50_ R/LD_50_ S)	LD_50_ resistance level	GR_50_ (g a.i. h^-1^)	RI (GR_50_ R/GR_50_ S)	GR_50_ resistance level
A	216.26 (7.22)	6.43	Low	154.91 (4.01)	6.35	Low
B	86.44 (0.72)	2.57	Low	128.97 (5.13)	5.29	Low
C	121.51 (2.94)	3.61	Low	42.61 (2.27)	1.75	Sensitive
S	33.63 (0)	-	Sensitive	24.40 (0)	-	Sensitive
MR220CL2	263.58 (3.47)	7.84	Moderate	191.12 (7.67)	7.83	Moderate
MR219	34.59 (0)	1.03	Sensitive	25.15 (0)	1.03	Sensitive

### Amplification and sequencing of the AHAS gene fragment

The primer pairs yielded the full-length 1884 base pair (bp) AHAS gene of *Oryza* sp. BLAST analysis revealed that the sequenced AHAS gene fragments were identical to the previously characterized *Oryza* AHAS gene available from the GenBank database, exhibiting 99.8% similarity. The amplified AHAS sequences have been deposited in GenBank (Accession number MN268688 for herbicide-resistant weedy rice and MN268687 for herbicide-susceptible weedy rice). Table 3 shows the nucleotide polymorphisms and amino acid substitutions observed in the AHAS gene sequences of R and S weedy rice populations, non-IMI- and IMI-rice varieties, and *Arabidopsis thaliana*. Four mutations were observed, at amino acid positions 93, 557, 653, and 669, of which the mutation at position 557 was a silent mutation. The mutations at positions 93, 557, and 669 lay outside the conserved domains, whereas that at position 653 was within Domain E. A silent mutation occurs when a codon is changed to another codon yielding the same amino acid [33]. We observed a silent mutation at the amino acid position 557, where the CCG codon in weedy rice and MR220CL2 was changed to CCT in MR219. Both CCG and CCT are redundant codons for proline, and thus, such a mutation does not alter the function of the overall protein.

**Table 3:** Nucleotide polymorphisms and amino acid substitutions in the AHAS sequences of *Arabidopsis thaliana*, weedy rice populations, and control rice varieties.

Amino acid position and relative sequence of nucleotide and amino acid				
Populations	93	557	653	669
*Arabidopsis thaliana*	CCA P	CCA P	AGT S	AAA K
Weedy rice R	CCG P	CCG P	AAT N	ATG M
Weedy rice S	CCG P	CCG P	AGT S	ATG M
MR220CL2	CCG P	CCG P	AAT N	ATG M
MR219	ACG T	CCT P	AGT S	GTG V

The sequenced AHAS gene from the R populations of weedy rice exhibited 99% amino acid sequence similarity with S weedy rice and MR219, and 100% similarity with MR220CL2. A single nucleotide polymorphism (SNP) at amino acid position 653 was revealed in the DNA sequence analysis, where G at the second base of the codon in S weedy rice was substituted by A in R weedy rice, resulting in the substitution of amino acid Serine (AGT) by Asparagine (AAT). A similar SNP was observed in the MR220CL2 AHAS gene, whereas no mutation at the Ser_653_ codon could be detected for MR219, similar to that in S weedy rice. The presence of AHAS Ser-653-Asn substitution was confirmed in all surviving R populations of weedy rice (populations A, B, and C) and MR220CL2 with DNA sequencing of at least three individuals, as described by Tardif et al. [34]. The chromatogram of the AHAS sequencing results revealed that the Ser-653-Asn mutation in all R weedy rice and MR220CL2 was homozygous (single peak) and located within the conserved Domain E of the AHAS gene (S1 Appendix).

### *In vitro* AHAS assay

**Fig 4:**
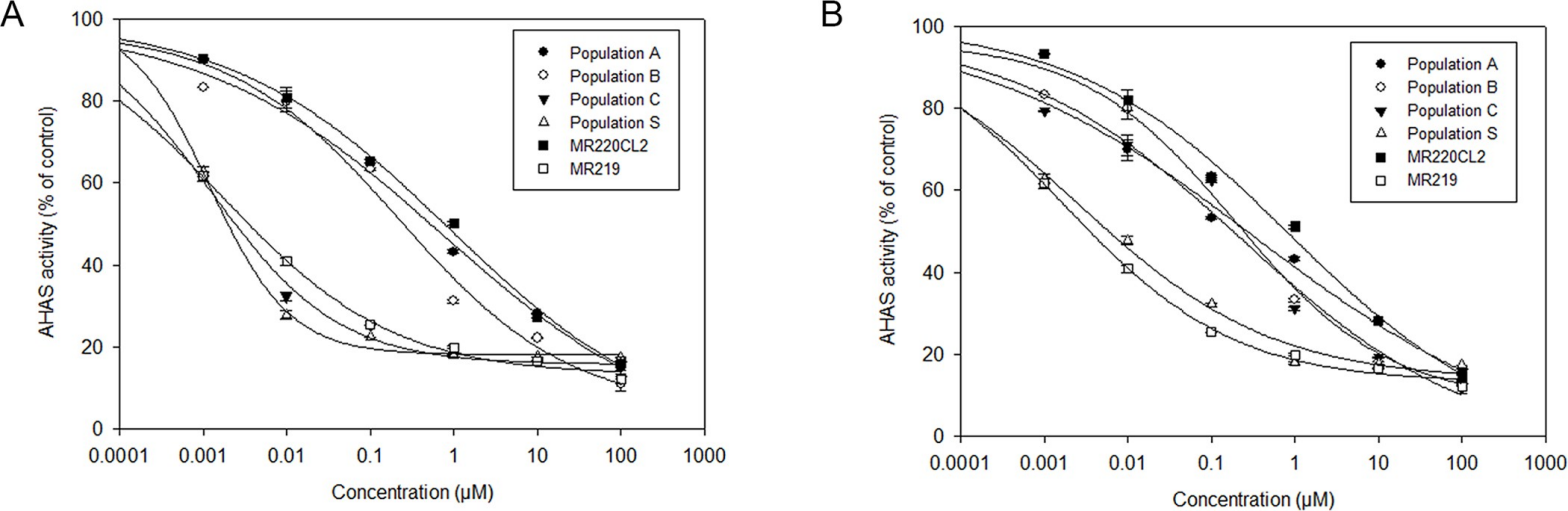
**A.** *In vitro* inhibition of AHAS activity by imazapic in weedy rice populations and control rice varieties. Data are expressed as means and standard errors of the mean, calculated from three extractions and four assays for each plant. **B.** *In vitro* inhibition of AHAS activity by imazapyr in weedy rice populations and control rice varieties. Data are expressed as means and standard errors of the mean, calculated from three extractions and four assays for each plant.

Overall, the AHAS *in vitro* assay for imazapic revealed that populations A, B, and MR220CL2 were considerably less sensitive to the herbicide than the weedy rice population C, S, and MR219 (Fig 4A). It was evident from the log-logistic curve of the imazapyr AHAS inhibition assay that the AHAS enzyme in all R populations of weedy rice (A, B, and C) and MR220CL2 was less sensitive to the herbicide, whereas S weedy rice and MR219 were equally susceptible (Fig 4B).

The I_50_ values for populations A and B treated with different concentrations of imazapic were 605- and 186-fold greater than that for S weedy rice, respectively (Table 4). As expected, MR220CL2 presented the highest RI value following imazapic treatment. Meanwhile, the AHAS enzyme in populations C, S, and MR219 was equally sensitive to imazapic application. The I_50_ values obtained from this inhibition assay revealed that the sensitivity of population C was similar to that of S weedy rice (0.0012), whereas MR219 was slightly less sensitive to imazapic than S (I_50_ = 0.0017). According to the RI values obtained from this experiment, the populations can be ranked from the most imazapic resistant to the least resistant as follows: MR220CL2 > A > B > MR219 ≥ C = S.

**Table 4:** Dose required to cause 50% inhibition of AHAS activity (I_50_), and the Resistance Index (RI) of weedy rice populations and control rice varieties.

Imidazolinone herbicides	Populations	AHAS activity (μmol acetoin formed mg protein^-1^ h^-1^)	I_50_ (μM)	RI (I_50_ R/I_50_ S)
Imazapic	A	68.83	0.7267	605.58
	B	62.55	0.2232	186.00
	C	65.27	0.0012	1.0
	S	24.22	0.0012	-
	MR220CL2	71.65	0.9062	755.17
	MR219	26.17	0.0017	1.42
Imazapyr	A	65.45	0.3352	134.08
	B	66.18	0.2450	77.16
	C	63.28	0.2398	95.92
	S	22.74	0.0025	-
	MR220CL2	78.65	0.8697	347.88
	MR219	22.98	0.0017	0.68

In the imazapyr AHAS inhibition assay, populations of R weedy rice (A, B, and C) were found to be 134, 77, and 95 times more resistant to imazapyr, respectively, than S weedy rice (Table 4). A lower concentration of imazapyr than imazapic was needed to achieve 50% inhibition of the AHAS activity in MR220CL2, although MR220CL2 exhibited the highest I_50_ RI value for imazapyr among the tested populations. The I_50_ for MR219 was 0.0012, indicating that the population was more sensitive to imazapyr than S weedy rice (I_50_ = 0.0025). Furthermore, population C was observed to be less sensitive to imazapyr than to imazapic. The rice populations could be ranked from the most imazapyr resistant to the least resistant as follows: MR220CL2 > A > C > B > S > MR219.

## Discussion

Petri dish seed bioassay provides a reliable and simple method to evaluate IMI resistance in weed populations [35]. Results from the seed bioassay in the present study indicated that continuous exposure of pre-germinated seeds to IMI herbicides can significantly affect seed growth. Variability in IMI herbicide sensitivity of weedy rice populations has also been observed in the USA [3, 36], Greece [28, 37], and Italy [38], as evident from the Petri dish seed bioassays. The seed bioassay results clearly indicated that in all weedy rice populations, the shoot was more sensitive to IMI herbicides than the root. Shivrain et al. [3] also found that the shoots of a US weedy rice accession treated with different concentrations of imazethapyr (another IMI herbicide) were more sensitive to the herbicide than the roots. However, exposure to IMI herbicides resulted in greater ‎inhibition of root growth in MR220CL2, and this differential effect of ‎IMI herbicides on weedy rice and MR220CL2 warrants further investigation. Roots and shoots are actively dividing meristematic tissues and the primary sites of AHAS expression [14]. The biosynthesis of branched-chain amino acids, i.e., valine, leucine, and isoleucine, mainly occurs in young tissues as these amino acids are crucial for the development of new cells, and thus, roots and shoots are the first to exhibit injury symptoms following herbicide inhibition [39]. It was observed that IMI herbicide treatment caused the seedling shoots to display signs of desiccation, whereas the inhibited hypocotyl turned black, eventually leading to plant death (Fig 2A and 2B).

In the whole-plant dose-response, all weedy rice populations presented low resistance to IMI herbicides. Similar results were obtained in a whole-plant dose-response experiment conducted in Brazil where low resistance was reported in weedy rice populations treated with a similar IMI herbicide premix of imazapic and imazapyr (weedy rice RI ranging from 3.5 to 4.5) [40]. Interestingly, population C exhibited a different resistance pattern to that of other resistant weedy rice populations and control rice varieties at the whole-plant level. Based on the LD_50_ value, population C possessed low herbicide resistance, which was contradictory to its observed sensitivity in the seed bioassay. It was noteworthy that the GR_50_ RI of population C was 1.75 (RI of 2 indicates low resistance). This was further supported by the I_50_ value, with population C exhibiting a more sensitive enzyme response to imazapic than to imazapyr (Table 4). If inappropriate application of IMI herbicides is continued, the imazapic–imazapyr resistance pattern is likely to shift and escalate in some weedy rice populations.

The seed bioassays facilitated the evaluation of the pre-emergence activity of the IMI herbicide premix on weedy rice populations and control rice varieties, whereas the whole-plant dose-response experiment allowed us to confirm the resistance of the populations to the post-emergence herbicide activity [41]. Hence, the two experiments, in conjunction, revealed the occurrence and level of resistance of weedy rice populations and control rice varieties to imazapic and imazapyr, which are most effective when applied as pre- and early post-emergence herbicides, respectively. The differences between the resistance patterns of populations A and B and population C may be attributed to improperly timed application of IMI herbicides in the affected areas. This was also reported in our recent interview with the rice growers of all 10 rice granaries in Peninsular Malaysia, where several farmers employing IMI herbicides admitted to delaying the application of IMI herbicides to 14 days after sowing (Ahmad-Hamdani, pers. comm.), which might have triggered the evolution of resistance to imazapyr in weedy rice populations (e.g., population C). Sartori et al. [42] opined that pre-emergence application of imazapic was effective in controlling weedy rice. A similar outcome was reported by Washburn and Barnes [43], who observed that pre-emergence application of imazapic was more effective in controlling grass weeds than post-emergence application. Mangold et al. [44] also reported that imazapic is more efficient in controlling downy brome (*Bromus tectorum* L.) seedlings when applied immediately after emergence, compared with application to older seedlings. However, imazapyr is more effective when applied post-emergence, even though it can be used both pre- and post-emergence [45]. The IMI herbicide premix used in this study (OnDuty™ WG, BASF Malaysia Sdn. Bhd.) contains more imazapic (52.5%) than imazapyr (17.5%) (3:1 ratio), both of which are the active ingredients. In the Malaysian IMI-rice Clearfield Stewardship, it is clearly stated that IMI herbicides must be applied pre- or early post-emergence, particularly between 0 and 5 days after sowing (when rice and weedy rice seedlings are at the 1–2-leaf stage), for effective weedy rice control. Hence, early application of IMI herbicides is crucial, and late application (12 days after sowing) can reduce its efficacy and expedite resistance evolution in weedy rice [16].

The MR220CL2 variety presented the highest LD_50_ value and moderate resistance to IMI herbicides at the whole-plant level (Table 2). MR220CL2 exhibited greater resistance to IMI herbicides in the seed bioassay than in the whole-plant dose-response, which is in agreement with the findings of Dilipkumar et al. [16]. Interestingly, even though this herbicide-resistant rice variety exhibited higher resistance to IMI herbicides than the weedy rice populations, a significant reduction in survival rate and shoot dry weight was recorded with herbicide application at 2 times the recommended rate (308 g a.i. ha^-1^) (Fig 3A and 3B). This shows that although MR220CL2 was moderately resistant to the IMI herbicides, its growth was impeded to a certain degree. Thus, it is important that farmers follow the guidelines on the application time of the IMI herbicides to ensure efficient weedy rice control and minimal injury to rice plants.

PCR amplification of plant samples from all tested populations and varieties yielded the entire AHAS gene fragment (1884 bp), containing all six conserved regions, as described by Merotto et al. [32]. The conserved regions C, A, D, F, B, and E, also known as domains, represent the gene regions where mutations endowing herbicide-resistance have previously been recorded. The comparison of AHAS gene fragment sequences from the weedy rice populations and control rice varieties indicated 99–100% amino acid sequence similarity. This proves that the AHAS gene sequences are highly conserved in *Oryza* spp., in both weedy and cultivated rice, at the nucleotide level in the AHAS coding region (Table 3). Rajguru et al. [46] stated that weedy and cultivated rice are expected to differ genetically at various loci, demonstrating the phenotypic plasticity of weedy species, especially in the unamplified regions. Although *Oryza* spp. generally exhibit low genetic variation within a population because of their self-pollinating nature, genetic variation can occur widely among populations [3, 47]. For instance, pollen-mediated gene flow and hybridization between cultivated rice and weedy rice have been evidenced in several studies [21–25]. Gene flow and hybridization events occurring among the *Oryza* spp. have been reported to contribute to genetic variation in weedy rice species of Malaysia [48]. A recent morphological study of Malaysian weedy rice provided evidence that Malaysian weedy rice have originated from wild *Oryza* species and modern-bred elite cultivars [49]. The 99% similarity among the AHAS gene sequences of weedy rice, S, and MR219 observed in this study indicates a close resemblance of the Malaysian weedy rice to its local cultivated rice variety.

The amino acid substitutions at positions 93 and 669, resulting in Pro_93_Thr and Val_669_Met, respectively, were evident in all weedy rice populations and MR220CL2. Shivrain et al. [3] recorded similar Pro-93-Thr and Val-669-Met mutations in weedy rice accessions from Arkansas, USA, with respect to the IMI herbicide-susceptible local rice cultivar Bengal. Pro_93_ and Val_669_ in the Bengal rice cultivar were substituted by Thr_93_ and Met_669_, respectively, as evident in the weedy rice populations and IMI-rice cultivars (Table 3). In addition, Sales et al. [22] recorded a similar Val-669-Met substitution in resistant red rice accessions Lon-2 and Ran-4 from the USA, when compared to the Bengal rice cultivar. Both these findings were consistent with the substitution of Val_669_ by Met_669_ in R and S weedy rice and MR220CL2 observed in this study. Nonetheless, considering that this mutation is outside the binding domains of AHAS inhibitors, the Val_669_Met substitution cannot be related to the reduced sensitivity of this enzyme to AHAS herbicides until a further study is conducted on the enzyme structure associated with this amino acid substitution. The results obtained in the present study further corroborate that the Val_669_Met substitution is not a resistance-endowing mutation as this substitution occurred in both R and S weedy rice populations (Table 3).

To date, a total of 27 amino acid substitutions in the AHAS gene at eight conserved positions conferring resistance to AHAS inhibitors in various weed species have been identified [11]. The eight conserved positions conferring resistance to IMI herbicides are Ala_122_, Pro_197_, Ala_205_, Asp_376_, Arg_377_, Trp_574_, Ser_653_, and Gly_654_ [11, 50]. In this study, a resistance-endowing polymorphism was observed at only one of the eight conserved positions, i.e., at amino acid position 653. It is known that substitution of G by A at the amino acid position 653, resulting in the change from Serine to Asparagine, is responsible for herbicide resistance in CPS technology [51, 52]. The Malaysian IMI-rice cultivars (MR220CL1 and MR220CL2) were derived from crosses between the Malaysian local rice variety MR220 and Louisiana State University (LSU) North America IMI-rice cultivar CL1770 harboring a Ser-653-Asn mutation [51]. Thus, the presence of Ser-653-Asn mutation in the AHAS gene of the Malaysian IMI-rice variety was confirmed through this study. The SNP causing the substitution at amino acid position 653 in IMI-rice was expected, as this is the mechanism of resistance to IMI herbicides in IMI-rice cultivars [21] and other species of weeds, including weedy rice [53].

The Ser_653_ mutation is of particular interest, as it is located in a highly conserved amino acid domain, and is commonly reported in plants with field-evolved resistance to AHAS inhibitors [32]. Tranel and Wright [10] summarized that changes in individual amino acids of the AHAS enzyme can convert a herbicide-susceptible plant to a herbicide-resistant plant, and Ser_653_, located at the C-terminal of the gene, is an important resistance-endowing mutation. A Ser-653-Thr substitution was reported in *Amaranthus powellii* S. Wats. that survived the application of imazethapyr and exhibited cross-resistance to atrazine [54]. Additionally, a Ser-653-Asn substitution was found in the imazethapyr-resistant *Amaranthus rudis* Sauer [55]. Ser_653_- mutations have also been widely reported in IMI herbicide-resistant weedy and red rice populations. An imazethapyr-resistant Strawhull hybrid red rice population in the southern United States was observed to harbor Ser-653-Thr mutation [21], whereas Ser-653-Asp and Ser-653-Asn mutations were reported in the IMI-resistant red rice samples collected during the 2006/2007 and 2007/2008 planting seasons in southern Brazil [56]. Busconi et al. [57] further observed a Ser-653-Asn mutation in red rice accessions resistant to the IMI herbicide imazamox in Italian Clearfield^®^ rice fields. It was subsequently found that all red rice populations in Italian Clearfield^®^ rice fields had developed resistance to imazamox, resulting from the same Ser to Asn substitution at locus 653 of the gene [58]. In addition, a homozygous mutation of Ser-653-Asn was recorded in both northern Greece putative resistant red rice and Clearfield^®^ rice cultivars, and PCR for ‘Clearfield_®_ allele’ detection proved that the genetic background of the putative resistant red rice matched that of the Clearfield^®^ rice cultivar [28].

The *in vitro* AHAS assay results of weedy rice populations and control rice varieties were similar to the results obtained in the seed bioassay and whole-plant dose-response experiments. However, the pattern of resistance conferred by the Ser-653-Asn mutation in Malaysian IMI-rice was slightly different from that in other IMI-rice with the same mutation, for example, Avila et al. [59] found that the CL-161 rice variety harboring the Ser-653-Asn mutation was 420 times more resistant to imazethapyr than its related S weedy rice ecotypes and the conventional rice variety Cypress. However, the RI values recorded in the *in vitro* enzyme assay of Malaysian IMI-rice revealed that this variety was 755 times more resistant to imazapic and 347 times more resistant to imazapyr, when compared with the S population. The variability in the IMI herbicide-resistance pattern at the enzyme level in populations carrying the same point mutation can be explained by the plant AHAS crystal structure. The difference in the chemical structures of AHAS inhibitors, even though from the same family, can influence the binding orientation of the herbicide to the target domain of the enzyme, with the IMI herbicide exhibiting different binding orientation [12, 50, 60]. This can also explain the variability in the resistance level of weedy rice populations to imazapic and imazapyr in this experiment. The RI values varied considerably across the populations under study, even though all surviving plants harbored the same Ser-653-Asn mutation.

To conclude, this study proves the occurrence of cross-resistance to the IMI herbicides imazapic and imazapyr in all weedy rice populations, resulting from an herbicide-insensitive AHAS enzyme, due to a Ser-653-Asn mutation in the AHAS gene. Populations A and B displayed greater resistance to imazapic, whereas population C also exhibited resistance to imazapyr. Future studies are needed to determine if the evolution of resistance in weedy rice populations across Malaysian IMI-rice fields is owing to gene flow from the IMI-rice to the weedy rice or spontaneous mutation resulting from continuous exposure of the weed to IMI herbicides. This study can serve as a reference to rice research institutes and agricultural policy decision makers of Malaysia if new herbicide-resistant rice were to be introduced in the future. Our findings could also aid the local agricultural-extension authorities in selecting different management approaches for resistant weedy rice populations, e.g., integration of the non-chemical such as cultural method for sustainable management of herbicide-resistant weeds.

## Supporting information

S1 Appendix DNA sequencing results showing (a) the AAT codon of Ser-653 in homozygous resistant plants, (b) the AGT codon of Ser-653 in a susceptible plant, (c) the AAT codon for Ser-653 in MR220CL2, and (d) the AGT codon for Ser-653 in MR219. Note: The lines represent Guanine (black), Adenine (green), Thymine (red), and Cytosine (blue).
(TIF)
