# Correction: Ser-653-Asn substitution in the acetohydroxyacid synthase gene confers resistance in weedy rice to imidazolinone herbicides in Malaysia

**DOI:** 10.1371/journal.pone.0244686

**Published:** 2020-12-22

**Authors:** Rabiatuladawiyah Ruzmi, Muhammad Saiful Ahmad-Hamdani, Norida Mazlan

There is information missing from the Funding statement. The complete, correct Funding statement is as follows: This research was funded by Universiti Putra Malaysia GP-IPS Research Grant (UPM/700-2/1/GP-IPS/2016/9492300) and Fundamental Research Grant Scheme under the Ministry of Higher Education Malaysia (FRGS/1/2018/WAB01/UPM/02/1: 07-01-18-1961FR; 5540086).
