# Supplementary figures and images for: Ser-653-Asn substitution in the acetohydroxyacid synthase gene confers resistance in weedy rice to imidazolinone herbicides in Malaysia
Source: PLoS One. 2020 Sep 14;15(9):e0227397. doi: 10.1371/journal.pone.0227397 (PMC7489537; doi:10.1371/journal.pone.0227397)

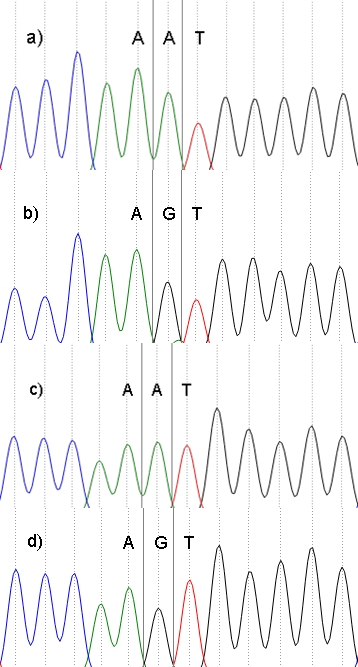

Supplement: S1 Appendix — DNA sequencing results showing (a) the AAT codon of Ser-653 in homozygous resistant plants, (b) the AGT codon of Ser-653 in a susceptible plant, (c) the AAT codon for Ser-653 in MR220CL2, and (d) the AGT codon for Ser-653 in MR219. Note: The lines represent Guanine (black), Adenine (green), Thymine (red), and Cytosine (blue). (TIF) [file pone.0227397.s001.tif]
